# Serum concentrations of phthalate metabolites are related to abdominal fat distribution two years later in elderly women

**DOI:** 10.1186/1476-069X-11-21

**Published:** 2012-04-02

**Authors:** P Monica Lind, Vendela Roos, Monika Rönn, Lars Johansson, Håkan Ahlström, Joel Kullberg, Lars Lind

**Affiliations:** 1 Occupational and Environmental Medicine, Uppsala University, Uppsala, Sweden; 2 Department of Radiology, Oncology and Radiation Science, Uppsala University, Uppsala, Sweden; 3 Department of Medicine, Uppsala University, Uppsala, Sweden

**Keywords:** Phthalates, Obesity, Mono-isobutyl phthalate, DXA, Abdominal MRI, PIVUS, Fat distribution, PPAR agonist

## Abstract

**Background:**

Phthalates, commonly used to soften plastic goods, are known PPAR-agonists affecting lipid metabolism and adipocytes in the experimental setting. We evaluated if circulating concentrations of phthalates were related to different indices of obesity using data from the Prospective Investigation of the Vasculature in Uppsala Seniors (PIVUS) study. Data from both dual-energy X-ray absorptiometry (DXA) and abdominal magnetic resonance imaging (MRI) were used.

**Methods:**

1,016 subjects aged 70 years were investigated in the PIVUS study. Four phthalate metabolites were detected in the serum of almost all subjects (> 96%) by an API 4000 liquid chromatograph/tandem mass spectrometer. Abdominal MRI was performed in a representative subsample of 287 subjects (28%), and a dual-energy X-ray absorptiometry (DXA)-scan was obtained in 890 (88%) of the subjects two year following the phthalate measurements.

**Results:**

In women, circulating concentrations of mono-isobutyl phthalate (MiBP) were positively related to waist circumference, total fat mass and trunk fat mass by DXA, as well as to subcutaneous adipose tissue by MRI following adjustment for serum cholesterol and triglycerides, education, smoking and exercise habits (all *p *< 0.008). Mono-methyl phthalate (MMP) concentrations were related to trunk fat mass and the trunk/leg-ratio by DXA, but less powerful than MiBP. However, no such statistically significant relationships were seen in men.

**Conclusions:**

The present evaluation shows that especially the phthalate metabolite MiBP was related to increased fat amount in the subcutaneous abdominal region in women measured by DXA and MRI two years later.

## Background

Phthalates (phthalate diesters) are commonly used as plasticisers in e.g. polyvinylchloride (PVC) plastics, and are therefore found in numerous household products such as food packaging, furniture, and toys; and in medical devices such as tubing and intravenous bags. In addition, phthalates are used in personal care products and pharmaceuticals. Since phthalates are additives and as such not covalently bound to the plastic, they can easily leach and transfer to e.g. air and food. Humans are exposed to phthalates through inhalation, ingestion, and dermal exposure, and exposure is ubiquitous due to the abundance of plastic in our society and in our homes. Phthalates are rapidly degraded into the respective phthalate monoesters in phase I reactions catalysed by lipases and esterases. The respective monoesters are eliminated in the urine as glucuronide conjugates or are further metabolised, and it is in fact the monoester metabolites that have been claimed to be responsible for adverse health effects [1-4].

Phthalates have been identified as endocrine-disrupting compounds (EDCs) with anti-androgenic effects on the developing male reproductive system through decreased testosterone biosynthesis [5,6]. Besides effects on reproduction, increasing attention is now being focused on the metabolic effects of phthalates and other EDCs, e.g. in the development of obesity [7-12]. The prevalence of obesity and associated diseases such as type 2 diabetes and cardiovascular diseases have risen dramatically worldwide during the last decades [13], and the search is on for explanations to complement the main causes of high caloric intake combined with low caloric expenditure, such as EDC exposure [11,14].

Several phthalate monoesters indeed bind to and activate the human peroxisome proliferator-activated receptors (PPARs) [15,16]. PPAR gamma is involved in fat storage by promoting adipocyte maturation and survival, and also controls insulin sensitivity [17]. PPAR gamma is targeted by the anti-diabetic thiazolidinedione drugs, whose agonistic actions not only improve insulin sensitivity but also promote weight gain [17,18]. Accordingly, PPARs activation by MEHP and other phthalate monoesters promotes the differentiation of both mouse and human preadipocytes into adipocytes [15,16,19].

In the present study, we evaluated if human serum phthalate monoester concentrations were related to different indices of obesity as determined using dual-energy X-ray absorptiometry (DXA) combined with abdominal magnetic resonance imaging (MRI). DXA provides estimates of lean and fat mass from a frontal whole-body scan, while the abdominal MRI employed determines visceral and subcutaneous adipose tissue areas in a single transversal section. Two previous studies have investigated the associations between urinary phthalate monoester concentrations and obesity using body mass index (BMI) and waist circumference (WC) in US citizens [9,10]. However, this is to our knowledge the first study on phthalate concentrations and obesity using advanced technology measures, allowing analyses of fat content in different body compartments (DXA), as well as comparisons between visceral and subcutaneous fat amount (MRI). We evaluated the hypothesis that phthalates could alter the adipose tissue distribution using measurements of fat distribution at MRI as our primary measurement. We also supply data on more commonly used obesity measures and DXA derived data.

## Methods

### Subjects

The study base for the PIVUS study (Prospective investigation of the vasculature in Uppsala seniors) were all subjects aged 70 (standard deviation for age 1 month, investigated 2001-2003) living in the community of Uppsala, Sweden. The subjects were randomly chosen from the register of community living. A total of 1,016 subjects participated, giving a participation rate of 50.1%. All women were postmenopausal. The study was approved by the Ethics Committee of Uppsala University.

All subjects were investigated in the morning after an overnight fast, since phthalate concentrations and lipid concentrations might change following a meal. No medication or smoking was allowed after midnight. The subjects were asked to answer a questionnaire about their medical history, education level, exercise habits, smoking habits, and regular medication. Education level was divided into three groups: <9 years, 9-12 years, and >12 years. Exercise habits were divided into four groups: <2 times light exercise (no sweat) per week, ≥2 times light exercise per week, 1-2 times heavy exercise (sweat) per week, and >2 times heavy exercise per week. Venous blood samples were collected and stored at -70°C until analysis of phthalate metabolites. Lipid variables were measured the same day as the blood was drawn using standard laboratory techniques. Basic characteristics of subjects are given in Table 1.

**Table 1:** Basic characteristics of subjects

	Women			Men		
**Variable**	**N**	**Mean (SD)**	**Median (25^th ^and 75^th ^percentile)**	**N**	**Mean (SD)**	**Median (25^th ^and 75^th ^percentile)**

BMI (kg/m^2^)	509	27.1 (4.9)	26.5 (23.7, 30.1)	507	27 (3.7)	26.8 (24.4, 29.1)

Waist (cm)	503	87.6 (11.6)	87.0 (79.0, 94.0)	501	94.7 (10.4)	94.0 (87.0, 101.0)

Waist/hip ratio	503	0.90 (0.10)	0.90 (0.80, 0.90)	501	0.90 (0.10)	0.9 (0.9, 1.0)

DXA total fat (kg)	459	27.7 (9.2)	27.1 (21.1, 33.7)	431	23.5 (8.2)	22.5 (17.6, 28.4)

DXA leg fat (kg)	459	9.7 (3.4)	9.4 (7.2, 11.8)	431	6.3 (2.6)	5.9 (4.7, 7.5)

DXA trunk fat (kg)	459	13.9 (5.0)	13.8 (10.2, 17.4)	431	14.1 (5.1)	13.8 (10.5, 17.5)

DXA trunk/leg ratio	459	1.5 (0.4)	1.4 (1.2, 1.7)	431	2.3 (0.6)	2.3 (1.9, 2.6)

MRI SAT (cm^2^)	139	262.1 (108.9)	257.4 (190.9, 313.9)	148	189.0 (80.3)	172.8 (131.3, 231.7)

MRI VAT (cm^2^)	139	94.3 (49.1)	87.5 (58.5, 119.2)	148	119.3 (63.0)	102.4 (79.2, 151.9)

MRI VAT/SAT ratio	139	0.4 (0.2)	0.3 (0.2, 0.5)	148	0.6 (0.3)	0.6 (0.5, 0.8)

MEHP (ng/ml)	502	18.9 (42.5)	4.7 (2.0, 14.5)	501	20.3 (45.0)	4.3 (2.1, 17.4)

MEP (ng/ml)	502	14.0 (10.3)	11.6 (7.2, 16.8)	501	18.0 (21.1)	11.6 (7.2, 18.5)

MiBP (ng(ml)	502	44.6 (116)	13.4 (9.5, 24.5)	501	53.6 (146.1)	13.5 (9.1, 33.3)

MMP (ng/ml)	502	3.4 (5.1)	1.5 (0.9, 3.0)	501	3.3 (4.5)	1.5 (0.8, 3.2)

Current smoking (%)	509	11.4		506	9.9	

Serum cholesterol (mmol/l)	507	5.7 (1.0)	5.7 (5.0, 6.3)	506	5.1 (1.0)	5.1 (4.5, 5.8)

Serum triglycerides (mmol/l)	508	1.3 (0.6)	1.1 (0.9, 1.5)	505	1.3 (0.6)	1.2 (0.9, 1.5)

Mean (SD) and median (25th and 75th percentile) given for the studied variables in women and men. The DXA and MRI measurements were performed two year following the measurements of phthalate metabolites and other measurements presented in the table

BMI-Body mass index; DXA-Dual-energy X-ray absorptiometry; MEHP-Mono-(2-ethylhexyl) phthalate; MEP-Mono-ethyl phthalate; MiBP-Mono-isobutyl phthalate; MMP-Mono-methyl phthalate; MRI-Magnetic resonance imaging; SAT-Subcutaneous adipose tissue; VAT-Visceral adipose tissue

### Phthalate analysis

Human serum (0.5 ml) was analysed for concentrations of ten phthalate metabolites by an API 4000 liquid chromatograph/tandem mass spectrometer at ALS Canada following the general procedures presented by the Centers for Disease Control and Prevention and as previously described [20]. Briefly, quality control of the analyses was maintained by analysing a method blank (calf serum) and two spiked calf serum samples (20 ng/ml, all analytes) along with every 17 samples. The detection limits (LOD), was 0.2 ng/ml. Four out of the ten phthalate metabolites, namely mono-(2-ethylhexyl) phthalate (MEHP); mono-ethyl phthalate (MEP); mono-isobutyl phthalate (MiBP); and mono-methyl phthalate (MMP), were detectable in all but 5-12 subjects (at least 96% of subjects). The fact that some subjects showed undetectable concentrations rules out a general contamination of these compounds. Only the four metabolites with detectable concentrations were used in the statistical analysis. For the rest of the metabolites, 31-100% of the observations were below the detection limit. The measured serum concentrations of MEHP, MEP, MiBP, and MMP are given in Table 1. The analysis of the phthalates took place 5-8 years following collection of the samples.

### Fat mass analysis by DXA

On average two years after the investigation at age 70 when data on phthalate metabolites were collected all subjects were invited to an investigation with dual-energy X-ray absorptiometry (DXA) (DPX, Lunar Prodigy, Lunar Corp., Madison, WI, USA), with the primary outcome total body fat mass. Of the original sample, 890 subjects (88% of subjects investigated at baseline) participated in this part of the study. Since abdominal fat repeatedly has been shown to be more harmful than fat located in the periphery [21] we also explored, as a secondary outcome, whether concentrations of phthalate metabolites were more closely related to trunk fat mass, defined as fat between the groins and the shoulders, or to leg fat mass, defined as fat below the groins.

In order to evaluate the reproducibility, fifteen subjects were scanned three times. The precision error of the DXA measurements was calculated to be 1.5% for total fat mass and 1.0% for total lean mass [22]. The bias associated with DXA fat measurement is systematic, with an underestimation of fat content for leaner subjects and an overestimation of fat content among obese subjects, but these inaccuracies amount to less than 2% [23].

### Abdominal MRI

Also magnetic resonance imaging (MRI) of the abdominal region was performed in 287 randomly selected subjects from the PIVUS cohort on average two years after the investigation at age 70 when data on phthalate metabolites were collected. Only a restricted sample could be offered this examination for financial reasons. Visceral and subcutaneous adipose tissue areas (cm^2^) (VAT and SAT, respectively) were manually segmented from a single 10 mm thick axial steady-state-free precession slice as previously described [24]. Based on repeated measurements of VAT and SAT in 22 subjects, the reproducibility/coefficients of variation (CVs) were found to be 5.9 and 3.4%, respectively.

Since the DXA and MRI scan measurements are not affected by a single over-night fast, these investigations were performed in the non-fasted state.

### Statistical analysis

All variables were evaluated regarding non-normality, and variables with a skewed distribution, such as plasma triglycerides and phthalate metabolite concentrations, were ln-transformed. In separate linear regression models for each phthalate metabolite and each gender, the phthalate metabolite concentrations (as ln-transformed continuous variables) were related to indices of obesity, as well as to fat mass measurements by DXA and abdominal MRI. In each model we adjusted for serum cholesterol and triglycerides, education, exercise, and smoking. In order to use the full power of the study, all subjects who had an obesity outcome measured were included in that particular model. The phthalate metabolites were also divided into quintiles to evaluate potential non-linear relationships, using both a linear and a quadratic term. For our primary hypothesis that the 4 phthalates were related to the two MRI measurements in both men and women, we adjusted the *p*-value for 16 tests (Bonferroni-adjusted *p*-value: 0.05/16 = 0.003125).

STATA 11 (College Station, TX, USA) was used for calculations.

## Results

### Basic characteristics

The basic characteristics of subjects and the serum concentrations of the four phthalate metabolites are presented in Table 1 as mean values (standard deviations), and as median concentrations (25^th ^and 75^th ^percentiles) in the total sample. No significant differences were seen in the subsample with DXA measurements, or in the subsample with MRI scans, when compared with the total sample.

### Linear regression analysis with continuous variable

The results from the linear regression analyses using circulating concentrations of phthalate metabolites as continuous variables are presented in Table 2 for women and in Table 3 for men. The results were adjusted for fasting serum cholesterol and triglycerides, education, smoking and exercise habits. In women, circulating concentrations of MiBP were positively related to WC, total fat mass and trunk fat mass by DXA, as well as to SAT by MRI (Table 2 all *p *< 0.008).

**Table 2:** Relationships between four phthalate metabolites and markers of obesity in women

		MEHP		MEP		MiBP		MMP	
**Variable**	**n**	**Beta (95% CI)**	***p*-value**	**Beta (95% CI)**	***p*-value**	**Beta (95% CI)**	***p*-value**	**Beta (95% CI)**	***p*-value**

BMI (kg/m^2^)	479	0.10 (-0.20-0.41)	0.51	0.008 (-0.67-0.69)	0.98	0.39 (0.002-0.79)	0.049	0.28 (-0.095-0.66)	0.14

Waist (cm)	474	0.34 (-0.39-1.1)	0.36	-0.80 (-2.4-0.81)	0.33	1.3 (0.425-2.3)	0.0043	0.98 (0.090-1.9)	0.031

Waist/hip ratio	474	0.002 (-0.002-0.006)	0.24	-0.008 (-0.016-0.001)	0.086	0.004 (0.000-0.009)	0.079	0.005 (0.000-0.009)	0.051

DXA total fat (kg)	431	468 (-159-1096)	0.14	-469 (-1877-938)	0.51	1079 (283-1875)	0.0082	705 (-74-1484)	0.077

DXA leg fat (kg)	431	163 (-73-399)	0.18	-165 (-694-363)	0.54	275 (-25-576)	0.0733	115 (-179-409)	0.44

DXA trunk fat (kg)	431	206 (-127-539)	0.23	-187 (-934-559)	0.62	699 (279-1120)	0.0012	546 (135-958)	0.0096

DXA trunk/leg ratio	431	-0.007 (-0.033-0.019)	0.60	-0.004 (-0.062-0.054)	0.88	0.039 (0.006-0.072)	0.021	0.043 (0.011-0.075)	0.0082

MRI SAT (cm^2^)	135	4.8 (-10-20)	0.53	12 (-24-48)	0.51	52 (24-81)	0.0004	27 (3.1-52)	0.029

MRI VAT (cm^2^)	135	4.1 (-2.2-10)	0.20	3.6 (-11-19)	0.63	14 (1.4-26)	0.031	6.9 (-3.4-17)	0.19

MRI VAT/SAT ratio	135	0.005 (-0.018-0.028)	0.67	-0.001 (-0.056-0.054)	0.96	-0.018 (-0.064-0.028)	0.45	-0.019 (-0.057-0.019)	0.34

Shown are results from linear regression models adjusting for serum cholesterol and triglycerides, education, exercise and smoking. The number of subjects used in the models for the different obesity outcome measurements are given as n. The units for the different obesity measures are given in table 1. The phthalate metabolites are given in ng/ml and ln-transformed values are used in the analysis

BMI-Body mass index; DXA-Dual-energy X-ray absorptiometry; MEHP-Mono-(2-ethylhexyl) phthalate; MEP-Mono-ethyl phthalate; MiBP-Mono-isobutyl phthalate; MMP-Mono-methyl phthalate; MRI-Magnetic resonance imaging; SAT-Subcutaneous adipose tissue; VAT-Visceral adipose tissue

**Table 3:** Relationships between four phthalate metabolites and markers of obesity in men

		MEHP		MEP		MiBP		MMP	
**Variable**	**n**	**Beta(95% CI)**	***p*-value**	**Beta (95% CI)**	***p*-value**	**Beta (95% CI)**	***p*-value**	**Beta (95% CI)**	***p*-value**

BMI (kg/m^2^)	482	-0.034 (-0.26-0.19)	0.77	0.31 (-0.097-0.72)	0.14	-0.083 (-0.35-0.19)	0.55	0.33 (0.051-0.61)	0.021

Waist (cm)	478	-0.27 (-0.91-0.37)	0.41	0.73 (-0.45-1.9)	0.22	-0.025 (-0.80-0.75)	0.95	0.97 (0.17-1.78)	0.0175

Waist/hip ratio	478	-0.004 (-0.008-0.0)	0.044	0.005 (-0.003-0.012)	0.22	0.001 (-0.004-0.005)	0.81	0.006 (0.001-0.011)	0.024

DXA total fat (kg)	410	12 (-527-552)	0.96	269 (-776-1315)	0.61	-73 (-754-608)	0.83	365 (-340-1071)	0.31

DXA leg fat (kg)	410	-24 (-200-152)	0.79	46 (-296-388)	0.79	-44 (-266-179)	0.70	37 (-194-268)	0.75

DXA trunk fat (kg)	410	0.56 (-332-333)	0.9974	347 (-297-991)	0.29	-23 (-443-398)	0.92	235 (-201-670)	0.29

DXA trunk/leg ratio	410	-0.008 (-0.048-0.033)	0.71	0.095 (0.018-0.17)	0.016	0.007 (-0.044-0.058)	0.78	0.021 (-0.031-0.074)	0.43

MRI SAT (cm^2^)	144	0.44 (-9.6-10)	0.93	9.2 (-10-29)	0.36	-11 (-34-12)	0.34	5.4 (-7.9-19)	0.43

MRI VAT (cm^2^)	144	3.3 (-4.9-11)	0.43	16 (0.49-32)	0.045	-5.9 (-24-13)	0.54	5.2 (-5.7-16)	0.35

MRI VAT/SAT ratio	144	0.002 (-0.032-0.036)	0.91	0.052 (-0.013-0.12)	0.12	0.001 (-0.076-0.079)	0.98	0.003 (-0.043-0.048)	0.91

Shown are results from linear regression models adjusting for serum cholesterol and triglycerides, education, exercise and smoking. The number of subjects used in the models for the different obesity outcome measurements are given as n. The units for the different obesity measures are given in table 1. The phthalate metabolites are given in ng/ml and ln-transformed values are used in the analysis

BMI-Body mass index; DXA-Dual-energy X-ray absorptiometry; MEHP-Mono-(2-ethylhexyl) phthalate; MEP-Mono-ethyl phthalate; MiBP-Mono-isobutyl phthalate; MMP-Mono-methyl phthalate; MRI-Magnetic resonance imaging; SAT-Subcutaneous adipose tissue; VAT-Visceral adipose tissue

The relationship between MiBP and SAT at MRI shows a *p*-value below the critical *p*-value obtained after correction for multiple testing. MMP concentrations in women were positively related to trunk fat mass and to trunk/leg-ratio by DXA (Table 2 both *p *< 0.01). However, no such significant relationships were seen in women for MEHP or MEP (Table 2), and no significant relationships were found in men for either metabolite (Table 3).

According to the regression models, a doubling of the MiBP concentrations corresponded to an increase of 45 cm^2 ^in SAT at MRI, 0.8 kg in trunk fat mass at DXA, and 1.5 cm in waist circumference in women. The corresponding effects of a doubling of MMP were 35 cm^2 ^in SAT at MRI, 0.4 kg in trunk fat at DXA, and 0.8 cm in waist circumference in women.

### Quintile analysis

The results from the quintile analysis approach are presented in Additional file 1: Tables S1-S8. Quintile analysis showed the same linear relationships for MiBP in women as described above using continuous data. In addition, positive linear relations were found between MEP and DXA trunk/leg ratio, as well as between MMP and WC and waist/hip ratio, all in men. Furthermore, the quintile analysis showed U-shaped relationships between MEP and SAT by MRI in women (*p *= 0.0098), as well as between MiBP and waist/hip-ratio in men (*p *= 0.0011).

Since there is some bias in the DXA estimations regarding fat mass (see methods section), we performed a secondary analysis in which the fat mass values for the lean subjects (BMI < 25 kg/m^2^) were multiplied by 1.02, and by 0.98 in the obese (BMI > 30 kg/m^2^). This attempt to compensate for the bias introduced by the DXA technique did however only have marginally effects on the analysis presented above.

## Discussion

The present study showed that serum concentrations of the phthalate metabolites MiBP and MMP were positively correlated to several obesity indices in women. In particular, a strong positive relation was found between MiBP concentrations and the amount of abdominal subcutaneous adipose tissue measured by DXA and MRI two years following the phthalate measurements were performed.

### Strengths of the study

Our study employed a hitherto unique combination of dual-energy X-ray absorptiometry (DXA) and abdominal magnetic resonance imaging (MRI), while previous studies of the relationship between phthalate concentrations and obesity have been limited to the use of body mass index (BMI) and waist circumference (WC) [9,10]. Our approach combining DXA and abdominal MRI offers a whole new level of resolution and specificity to different aspects of obesity, in particular the distinction between visceral and subcutaneous adipose tissue, both as an MRI VAT/SAT ratio and as a DXA trunk/leg fat ratio. Furthermore, if we find a relationship between a phthalate metabolite and similar obesity measurements both at DXA and MRI, that will reduce the chance of false positive findings.

Both previous studies of phthalate concentrations in relation to obesity were based on data from the US National Health and Nutrition Examination Survey (NHANES) [9,10]. Neither study included MiBP or MMP. Stahlhut et al found positive associations between MEP concentrations and WC in men over 18 years of age [10], while Hatch et al found mixed associations between MEP/MEHP and BMI in women and men of different age groups [9]. Hatch et al also found negative relations between MEHP urine concentrations and BMI in both women and men aged 60-80 years [9], which was not observed in our subjects. The advantage with the PIVUS study compared to NHANES is that we could characterize obesity in more detail by regional distribution of fat mass by DXA and MRI.

### Limitations of the study

Two limitations in the present study related to the DXA and MRI measurements are that these examinations were performed two years following the measurements of the phthalates, and the fact that not all subjects investigated at baseline were investigated with the two imaging techniques. Regarding the time lag between the phthalate measurements and the imaging, this could lead to false negative results since both changes in phthalates and obesity measures could have occurred during the two year period that might have influenced the investigated relationships. However, it is very unlikely that the reported main finding that MiBP concentrations are related to trunk fat at DXA and to SAT at MRI could be created by a time lag. The fact that MiBP concentrations also were related to waist circumference, measured at the same day as MiBP, further strengthens the case that the main findings in the present study are not erroneously created by this time lag. However, only a true longitudinal study with serial evaluations with DXA and MRI will clarify this issue.

The fact that a time lag of two years will create a selection of survivors and a drop-out of subjects not willing to be reinvestigated is undisputed. We do not think that this selection of subjects attending the imaging procedures will introduce any major bias that will influence the results since all basic characteristics were essentially the same in the total sample and the subsamples attending the DXA and MRI scans.

In the present study we used serum measurements of phthalate metabolites. It is more common to use urinary measurements. The advantage of urinary measurements is that usually higher concentrations are found compared to serum and thereby more phthalate metabolites could be quantified above the lower detection limit. Therefore we can only report associations regarding four metabolites, although in fact 10 metabolites were evaluated. Serum concentrations might also change more rapidly than urinary concentrations, and therefore repeated measurements would be desirable for a more precise measure of exposure.

The participating subjects were all elderly (aged 70) Caucasians, which obviously confers limitations as to the applicability of the results with respect to the general population. For example, Hatch et al found large differences in associations between urine concentrations of phthalate metabolites and obesity depending on age group [9], and Stahlhut et al observed differences between ethnic groups [10]. On the other hand, the relative homogeneity and large number of subjects permit the detection of more subtle effects. Moreover, the age of our subjects isolates the effects of adult exposure and precludes any effects from gestational and perinatal phthalate exposure since these chemicals were not in use when the subjects were born. Furthermore, the populations in Europe and US are steadily increasing in age and therefore it is of importance to study the effect of exposure to environmental contaminants not only in the newborn, but also in the aged population.

### Gender differences and possible mechanisms

In this study, circulating concentrations of MiBP were positively related to several obesity indices in women, most notably trunk fat by DXA and SAT by MRI, while no such associations were seen in men. This gender difference is particularly interesting since no significant differences in phthalate metabolite concentrations were found between men and women in this material [25]. The differences in effects thus cannot be attributed to differences in phthalate concentrations, but may instead reflect gender-specific regulation of adipose tissue distribution. Using BMI and WC, Hatch et al found no gender differences in subjects aged 60-80 years [9], reflecting the higher precision of our DXA/MRI approach.

Several studies have described a cross-talk between estrogen or estrogen receptors and activation of PPAR receptors, the most well characterized target for the phthalates [26-28]. Ovariectomized rats treated with a PPAR alpha agonist showed a less pronounced increase in fat mass. This condition was reversed by estrogen treatment, which also reduced PPAR alpha expression [28]. Similarly, overiectomized mice treated with a PPAR gamma agonist showed a less pronounced increase in fat mass. Also this condition was reversed by estrogen treatment, which also reduced PPAR gamma expression [26]. Ovariectomy increased fat tissue in mice compared to sham-operated controls. This effect was inhibited in mice lacking the PPAR alpha gene [16]. Thus, if these experimental data suggesting a cross-talk between PPAR receptor activation and estrogen are similar in humans they may explain why activators of PPARs, such as phthalates, could have different effects in men and women.

As noted in the introduction, several phthalate monoesters have been identified as PPAR gamma agonists and capable of promoting adipocyte maturation, providing a plausible mechanistic link to phthalate effects on obesity development and adipose tissue distribution [8,15,16,19]. To our knowledge, MiBP has not been tested for PPAR activation, however MMP was found not to activate either PPAR alpha or PPAR gamma [16].

### Statistical considerations

Some non-linear relationships were noted in the quintile analysis, including some relationships in men. Non-monotonic relationships have been previously described for endocrine disruptors [29,30], however in this case the non-linear relationships were seen only for certain phthalates and certain obesity phenotypes, while MiBP was related to many obesity phenotypes in women in a consistent manner. The non-linear relationships described in the present study must thus be interpreted with caution.

In the present study, several statistical tests were performed and this could lead to false positive results due to multiple testing. However, regarding our primary hypothesis that the four phthalates were related to fat distribution measures at MRI a strict Bonferroni correction was used and when applying this MiBP was found to be related to SAT in women. All other tests were performed to seek for consistency between different obesity measures and are therefore not subjected to any adjustment for multiple testing. These results must therefore be taken with caution, but as could be seen these results fits well with findings regarding the primary hypothesis.

In the study we had outcome measures with variable number of observations, only one third as many MRI scans as DXA scans. Thus, when interpreting the *p*-values it must be kept in mind that an association has to be considerable stronger for a phthalate metabolite vs an obesity measured at MRI than vs an obesity measure at DXA to obtain a significant *p*-value. It is however not reasonable to delete two thirds of the entire sample from the calculations in order to make the *p*-values comparable, since that approach will induce false negative findings regarding the relationships between phthalate concentrations and obesity measurements at DXA.

## Conclusions

The present evaluation shows that especially the phthalate metabolite MiBP was related to increased fat amount in the subcutaneous abdominal region in women measured by DXA and MRI two years later.

## Abbreviations

BMI: Body mass index; DEHP: Di-ethylhexyl phthalate; DXA: Dual-energy X-ray absorptiometry; EDC: Endocrine-disrupting compound; MEHP: Mono-(2-ethylhexyl) phthalate; MEP: Mono-ethyl phthalate; MiBP: Mono-isobutyl phthalate; MMP: Mono-methyl phthalate; MRI: Magnetic resonance imaging; PIVUS: Prospective Investigation of the Vasculature in Uppsala seniors; PPAR: Peroxisome proliferator-activated receptor; SAT: Subcutaneous adipose tissue; VAT: Visceral adipose tissue; WC: Waist circumference.

## Competing interests

The authors declare that they have no competing interests.

## Authors' contributions

PML, VR and LL drafted the manuscript. VR contributed to critical revision of the manuscript for important intellectual content and in addition finalized the manuscript. MR contributed to critical revision of the manuscript for important intellectual content. PML conceived of, designed, and coordinated the study and contributed to critical revision of the manuscript for important intellectual content. LL, together with PML, conceived of, designed, and coordinated the study and contributed to critical revision of the manuscript for important intellectual content. Also, LL is principal investigator of PIVUS and had full access to all the data in the study and takes responsibility for the integrity of the data and the accuracy of the data analysis. JK, LJ and HA were responsible for the MRI laboratory analyses of VAT/SAT measurements and performed, acquisition, analysis and interpretation of this data and contributed to critical revision of the manuscript for important intellectual content. All authors read and approved the final manuscript.

## Supplementary Material

Additional file 1 **Table S1**. Mean and SD for different indices of obesity given in quintiles of the phthalate metabolite MEHP in women. **Table S2**. Mean and SD for different indices of obesity given in quintiles of the phthalate metabolite MEHP in men. **Table S3**. Mean and SD for different indices of obesity given in quintiles of the phthalate metabolite MEP in women. **Table S4**. Mean and SD for different indices of obesity given in quintiles of the phthalate metabolite MEP in men. **Table S5**. Mean and SD for different indices of obesity given in quintiles of the phthalate metabolite MiBP in women. **Table S6**. Mean and SD for different indices of obesity given in quintiles of the phthalate metabolite MiBP in men. **Table S7**. Mean and SD for different indices of obesity given in quintiles of the phthalate metabolite MMP in women. **Table S8**. Mean and SD for different indices of obesity given in quintiles of the phthalate metabolite MMP in men. Click here for file

## References

[B1] Wittassek M Angerer J Phthalates: metabolism and exposure Int J Androl 2008 31 2 131 138 10.1111/j.1365-2605.2007.00837.x 18070048

[B2] Koch HM Preuss R Angerer J Di(2-ethylhexyl)phthalate (DEHP): human metabolism and internal exposure-- an update and latest results Int J Androl 2006 29 1 155 165 discussion 181-155 10.1111/j.1365-2605.2005.00607.x 16466535

[B3] Frederiksen H Skakkebaek NE Andersson A-M Metabolism of phthalates in humans Molecular Nutrition & Food Research 2007 51 7 899 911 10.1002/mnfr.200600243 17604388

[B4] Heudorf U Mersch-Sundermann V Angerer J Phthalates: toxicology and exposure International journal of hygiene and environmental health 2007 210 5 623 634 10.1016/j.ijheh.2007.07.011 17889607

[B5] Foster PMD Disruption of reproductive development in male rat offspring following in utero exposure to phthalate esters International Journal of Andrology 2006 29 1 140 147 10.1111/j.1365-2605.2005.00563.x 16102138

[B6] Shultz VD Phillips S Sar M Foster PMD Gaido KW Altered Gene Profiles in Fetal Rat Testes after in Utero Exposure to Di(n-butyl) Phthalate Toxicological Sciences 2001 64 2 233 242 10.1093/toxsci/64.2.233 11719706

[B7] Grün F Blumberg B Perturbed nuclear receptor signaling by environmental obesogens as emerging factors in the obesity crisis Reviews in endocrine & metabolic disorders 2007 8 2 161 171 10.1007/s11154-007-9049-x 17657605

[B8] Desvergne B Feige JN Casals-Casas C PPAR-mediated activity of phthalates: a link to the obesity epidemic? Mol Cell Endocrinol 2009 304 1-2 43 48 10.1016/j.mce.2009.02.017 19433246

[B9] Hatch EE Nelson JW Qureshi MM Weinberg J Moore LL Singer M Webster TF Association of urinary phthalate metabolite concentrations with body mass index and waist circumference: a cross-sectional study of NHANES data, 1999-2002 Environmental health: a global access science source 2008 7 27 10.1186/1476-069X-7-27 18522739 PMC2440739

[B10] Stahlhut RW van Wijngaarden E Dye TD Cook S Swan SH Concentrations of urinary phthalate metabolites are associated with increased waist circumference and insulin resistance in adult U.S. males Environ Health Perspect 2007 115 6 876 882 10.1289/ehp.9882 17589594 PMC1892109

[B11] Newbold RR Impact of environmental endocrine disrupting chemicals on the development of obesity Hormones (Athens) 2010 9 3 206 217 20688618 10.14310/horm.2002.1271

[B12] Teitelbaum SL Mervish N LM E Vangeepuram N Galvez MP Calafat AM Silva MJ LB B Wolff MS Associations between phthalate metabolite urinary concentrations and body size measures in New York City children Environmental research 2012 112 186 193 22222007 10.1016/j.envres.2011.12.006 PMC3267869

[B13] Yach D Stuckler D Brownell KD Epidemiologic and economic consequences of the global epidemics of obesity and diabetes Nature medicine 2006 12 1 62 66 10.1038/nm0106-62 16397571

[B14] Neel BA Sargis RM The paradox of progress: environmental disruption of metabolism and the diabetes epidemic Diabetes 2011 60 7 1838 1848 10.2337/db11-0153 21709279 PMC3121438

[B15] Feige JN Gelman L Rossi D Zoete V Metivier R Tudor C Anghel SI Grosdidier A Lathion C Engelborghs Y The endocrine disruptor monoethyl-hexyl-phthalate is a selective peroxisome proliferator-activated receptor gamma modulator that promotes adipogenesis The Journal of biological chemistry 2007 282 26 19152 19166 10.1074/jbc.M702724200 17468099

[B16] Hurst CH Waxman DJ Activation of PPARα and PPARγ by Environmental Phthalate Monoesters Toxicological Sciences 2003 74 2 297 308 10.1093/toxsci/kfg145 12805656

[B17] Lehrke M Lazar MA The Many Faces of PPARγ Cell 2005 123 6 993 999 10.1016/j.cell.2005.11.026 16360030

[B18] Schwartz S Raskin P Fonseca V Graveline JF Effect of Troglitazone in Insulin-Treated Patients with Type II Diabetes Mellitus New England Journal of Medicine 1998 338 13 861 866 10.1056/NEJM199803263381302 9516220

[B19] Ellero-Simatos S Claus SP Benelli C Forest C Letourneur F Cagnard N Beaune PH de Waziers I Combined transcriptomic-^1^H-NMR metabonomic study reveals that monoethylhexyl phthalate stimulates adipogenesis and glyceroneogenesis in human adipocytes Journal of proteome research 2011 10 12 5493 502 [Online 200721 October 202011] 22017230 10.1021/pr200765v PMC3229183

[B20] Olsen L Lampa E Birkholz DA Lind L Lind PM Circulating levels of bisphenol A (BPA) and phthalates in an elderly population in Sweden, based on the Prospective Investigation of the Vasculature in Uppsala Seniors (PIVUS) Ecotoxicology and environmental safety 2012 75 1 242 248 21955883 10.1016/j.ecoenv.2011.09.004

[B21] Larsson B Svardsudd K Welin L Wilhelmsen L Bjorntorp P Tibblin G Abdominal adipose tissue distribution, obesity, and risk of cardiovascular disease and death: 13 year follow up of participants in the study of men born in 1913 Br Med J (Clin Res Ed) 1984 288 6428 1401 1404 10.1136/bmj.288.6428.1401 PMC1441047 6426576

[B22] Ingelsson E Sundström J Melhus H Michaëlsson K Berne C Vasan RS Risérus U Blomhoff R Lind L Ärnlöv J Circulating retinol-binding protein 4, cardiovascular risk factors and prevalent cardiovascular disease in elderly Atherosclerosis 2009 206 1 239 244 10.1016/j.atherosclerosis.2009.02.029 19339013

[B23] Wang Z Heymsfield SB Chen Z Zhu S Pierson RN Estimation of percentage body fat by dual-energy x-ray absorptiometry: evaluation by in vivo human elemental composition Physics in medicine and biology 2010 55 9 2619 2635 10.1088/0031-9155/55/9/013 20393230 PMC2921899

[B24] Kullberg J von Below C Lonn L Lind L Ahlstrom H Johansson L Practical approach for estimation of subcutaneous and visceral adipose tissue Clinical physiology and functional imaging 2007 27 3 148 153 10.1111/j.1475-097X.2007.00728.x 17445065

[B25] Olsén L Lampa E Birkholz DA Lind L Lind PM Circulating levels of bisphenol A (BPA) and phthalates in an elderly population in Sweden, based on the Prospective Investigation of the Vasculature in Uppsala Seniors (PIVUS) Ecotoxicology and environmental safety in press 10.1016/j.ecoenv.2011.09.004 21955883

[B26] Jeong S Yoon M 17beta-Estradiol inhibition of PPARgamma-induced adipogenesis and adipocyte-specific gene expression Acta pharmacologica Sinica 2011 32 2 230 238 10.1038/aps.2010.198 21293475 PMC4009938

[B27] Kim BH Won YS Kim DY Kim B Kim EY Yoon M Oh GT Signal crosstalk between estrogen and peroxisome proliferator-activated receptor alpha on adiposity BMB reports 2009 42 2 91 95 10.5483/BMBRep.2009.42.2.091 19250609

[B28] Jeong S Yoon M Inhibition of the actions of peroxisome proliferator-activated receptor alpha on obesity by estrogen Obesity (Silver Spring) 2007 15 6 1430 1440 10.1038/oby.2007.171 17557980

[B29] Lee DH Steffes MW Sjödin A Jones RS Needham LL Jacobs DR Jr Low dose organochlorine pesticides and polychlorinated biphenyls predict obesity, dyslipidemia, and insulin resistance among people free of diabetes PloS one 2011 6 1 e15977 10.1371/journal.pone.0015977 21298090 PMC3027626

[B30] Lee D-H Steffes MW Sjödin A Jones RS Needham LL Jacobs DR Jr Low Dose of Some Persistent Organic Pollutants Predicts Type 2 Diabetes: A Nested Case-Control Study Environ Health Perspect 2010 118 9 1235 1242 10.1289/ehp.0901480 20444671 PMC2944083

