# Supplementary material for: Serum concentrations of phthalate metabolites are related to abdominal fat distribution two years later in elderly women
Source: Environ Health. 2012 Apr 2;11:21. doi: 10.1186/1476-069X-11-21 (PMC3379932; doi:10.1186/1476-069X-11-21)
Supplement: Additional file 1 — Table S1. Mean and SD for different indices of obesity given in quintiles of the phthalate metabolite MEHP in women. Table S2. Mean and SD for different indices of obesity given in quintiles of the phthalate metabolite MEHP in men. Table S3. Mean and SD for different indices of obesity given in quintiles of the phthalate metabolite MEP in women. Table S4. Mean and SD for different indices of obesity given in quintiles of the phthalate metabolite MEP in men. Table S5. Mean and SD for different indices of obesity given in quintiles of the phthalate metabolite MiBP in women. Table S6. Mean and SD for different indices of obesity given in quintiles of the phthalate metabolite MiBP in men. Table S7. Mean and SD for different indices of obesity given in quintiles of the phthalate metabolite MMP in women. Table S8. Mean and SD for different indices of obesity given in quintiles of the phthalate metabolite MMP in men. [file 1476-069X-11-21-S1.PDF]

Table S1. Mean and SD for different indices of obesity given in quintiles of the phthalate metabolite MEHP in women.

	**Q1**	**Q2**	**Q3**	**Q4**	**Q5**		
**Variable**	**Mean (SD)**	**Mean (SD)**	**Mean (SD)**	**Mean (SD)**	**Mean (SD)**	**p-value linear**	**p-value quadratic**
BMI (kg/m^2)	26. 6 (4.2)	26.6 (5.1)	27.3 (5.4)	27.7 (5.1)	27.2 (4.6)	0.24	0.49
Waist (cm)	86.9 (10.7)	86.7 (12.1)	87.7 (11.7)	88.2 (11.9)	88.5 (11.5)	0.30	0.88
Waist/hip ratio	0.86 (0.06)	0.86 (0.07)	0.87 (0.06)	0.86 (0.06)	0.87 (0.06)	0.43	0.27
DXA total fat (kg)	26.3 (8.9)	26.5 (9.7)	28.0 (9.1)	29.1 (9.5)	28.2 (8.8)	0.056	0.43
DXA leg fat (kg)	9.2 (3.2)	9.4 (3.7)	9.7 (3.4)	10.2 (3.4)	10.0 (3.3)	0.062	0.60
DXA trunk fat (kg)	13.4 (4.9)	13.3 (5.1)	14.2 (4.9)	14.7 (5.2)	14.1 (4.7)	0.11	0.43
DXA trunk/leg ratio	1.48 (0.38)	1.46 (0.46)	1.50 (0.41)	1.47 (0.41)	1.43 (0.34)	0.52	0.61
MRI SAT (cm^2)	255.1 (88.8)	255.1 (124.5)	247.5 (93.1)	278.3 (131.8)	264.3 (92.0)	0.55	0.90
MRI VAT (cm^2)	90.6 (38.0)	90.5 (50.7)	93.3 (46.5)	94.6 (58.0)	97.8 (44.1)	0.57	0.72
MRI VAT/SAT ratio	0.36 (0.11)	0.37 (0.16)	0.41 (0.25)	0.34 (0.14)	0.38 (0.17)	0.78	0.95

Table S2. Mean and SD for different indices of obesity given in quintiles of the phthalate metabolite MEHP in men.

	**Q1**	**Q2**	**Q3**	**Q4**	**Q5**		
**Variable**	**Mean (SD)**	**Mean (SD)**	**Mean (SD)**	**Mean (SD)**	**Mean (SD)**	**p-value linear**	**p-value quadratic**
BMI (kg/m^2)	27.1 (3.7)	26.7 (3.5)	27.7 (3.7)	26. 7 (4.0)	26.7 (3.6)	0.68	0.90
Waist (cm)	94. 8 (10.5)	94.3 (10.2)	96.8 (10.9)	93.9 (10.9)	93. 7 (9.8)	0.45	0.47
Waist/hip ratio	0.95 (0.06)	0.95 (0.07)	0.95 (.07)	0.94 (0.06)	0.94 (0.07)	0.033	0.80
DXA total fat (kg)	23.4 (8.1)	22.7 (7.5)	24.9 (9.1)	22.7 (9.0)	23.3 (7.4)	0.999	0.99
DXA leg fat (kg)	6.5 (3.3)	6.0 (2.2)	6.7 (2.8)	6.0 (2.5)	6.4 (2.3)	0.75	0.50
DXA trunk fat (kg)	14.1 (5.1)	13.8 (4.7)	15.0 (5.5)	13.9 (5.8)	13.9 (4.6)	0.89	0.91
DXA trunk/leg ratio	2.34 (0.69)	2.32 (0.59)	2.30 (0.57)	2.36 (0.63)	2.23 (0.51)	0.37	0.84
MRI SAT (cm^2)	188.1 (84.0)	187.2 (88.1)	203.5 (84.6)	179.8 (81.6)	186.4 (70.1)	0.60	0.86
MRI VAT (cm^2)	96.2 (27.2)	118.2 (49.2)	122.8 (53.0)	118.7 (67.6)	120.0 (79.8)	0.19	0.49
MRI VAT/SAT ratio	0.59 (0.25)	0.66 (0.25)	0.63 (0.25)	0.66 (0.23)	0.64 (0.29)	0.84	0.59

Table S3. Mean and SD for different indices of obesity given in quintiles of the phthalate metabolite MEP in women.

	**Q1**	**Q2**	**Q3**	**Q4**	**Q5**		
**Variable**	**Mean (SD)**	**Mean (SD)**	**Mean (SD)**	**Mean (SD)**	**Mean (SD)**	**p-value linear**	**p-value quadratic**
BMI (kg/m^2)	27.4 (4.8)	27.2 (5.3)	26.9 (5.2)	26.5 (4.0)	27.8 (5.0)	0.60	0.083
Waist (cm)	89. 4 (11.6)	87.4 (12.3)	87.2 (12.1)	86.1 (10.3)	88.5 (11.5)	0.16	0.060
Waist/hip ratio	0.88 (0.06)	0.86 (0.05)	0.86 (0.06)	0.86 (0.07)	0.86 (0.06)	0.044	0.033
DXA total fat (kg)	28.4 (9.1)	28.4 (10.7)	26.5 (9.1)	27.1 (8.5)	28.2 (8.8)	0.48	0.20
DXA leg fat (kg)	9.8 (3.5)	10.0 (4.0)	9.6 (3.4)	9.5 (3.0)	9.8 (3.2)	0.50	0.72
DXA trunk fat (kg)	14.4 (4.8)	14.2 (5.7)	13.1 (4.9)	13.7 (4.7)	14.4 (4.7)	0.59	0.073
DXA trunk/leg ratio	1.53 (0.43)	1.46 (0.41)	1.40 (0.43)	1.47 (0.35)	1.51 (0.37)	0.87	0.028
MRI SAT (cm^2)	280.3 (94.6)	261.0 (136.0)	222.2 (85.5)	268.9 (97.6)	315.5 (120.8)	0.65	0.0098
MRI VAT (cm^2)	92.0 (34.2)	97.9 (63.1)	87.4 (52.9)	98.0 (42.8)	99.1 (43.5)	0.77	0.63
MRI VAT/SAT ratio	0.34 (0.12)	0.40 (0.25)	0.39 (0.18)	0.39 (0.16)	0.33 (0.12)	0.96	0.14

Table S4. Mean and SD for different indices of obesity given in quintiles of the phthalate metabolite MEP in men.

	**Q1**	**Q2**	**Q3**	**Q4**	**Q5**		
**Variable**	**Mean (SD)**	**Mean (SD)**	**Mean (SD)**	**Mean (SD)**	**Mean (SD)**	**p-value linear**	**p-value quadratic**
BMI (kg/m^2)	26. 7 (3.4)	27.0 (4.0)	26.6 (3.9)	27.6 (3.5)	27.0 (3.7)	0.25	0.60
Waist (cm)	93.9 (10.7)	94.8 (10.2)	94.5 (11.1)	96.5 (10.7)	93.9 (9.7)	0.44	0.19
Waist/hip ratio	0.94 (0.07)	0.95 (0.06)	0.95 (0.07)	0.95 (0.06)	0.94 (0.07)	0.41	0.28
DXA total fat (kg)	24.0 (8.8)	22.5 (8.3)	23.3 (9.1)	24.4 (7.5)	22.9 (7.4)	0.71	0.93
DXA leg fat (kg)	6.7 (2.8)	6.0 (2.6)	6.3 (2.5)	6.5 (2.3)	6.2 (3.0)	0.77	0.69
DXA trunk fat (kg)	14.2 (5.2)	13.6 (5.2)	14.1 (5.8)	14.9 (4.6)	14.0 (4.7)	0.28	0.81
DXA trunk/leg ratio	2.16 (0.56)	2.3 (0.63)	2.26 (0.60)	2.36 (0.51)	2.43 (0.64)	0.0033	0.91
MRI SAT (cm^2)	189.9 (104.4)	185.9 (87.3)	177.0 (67.6)	207.0 (73.6)	193. 6 (71.9)	0.35	0.77
MRI VAT (cm^2)	101.7 (48.7)	119.2 (67.9)	111.0 (69. 7)	131.9 (54.9)	134.3 (58.1)	0.056	0.61
MRI VAT/SAT ratio	0.57 (0.19)	0.65 (0.28)	0.63 (0.25)	0.67 (0.25)	0.72 (0.27)	0.16	0.997

Table S5. Mean and SD for different indices of obesity given in quintiles of the phthalate metabolite MiBP in women.

	**Q1**	**Q2**	**Q3**	**Q4**	**Q5**		
**Variable**	**Mean (SD)**	**Mean (SD)**	**Mean (SD)**	**Mean (SD)**	**Mean (SD)**	**p-value linear**	**p-value quadratic**
BMI (kg/m^2)	26.2 (4.3)	27. 1 (5.1)	26.8 (4.7)	28.0 (5.4)	27.3 (4.6)	0.021	0.55
Waist (cm)	85.0 (11.1)	87.3 (11.4)	86.4 (11.0)	90.0 (12.5)	89.6 (11.2)	0.0014	0.996
Waist/hip ratio	0.86 (0.07)	0.86 (0.06)	0.85 (0.07)	0.87 (0.06)	0.87 (0.06)	0.11	0.30
DXA total fat (kg)	25.5 (9.3)	27.7 (9.3)	27.5 (8.5)	28.6 (9.4)	29.0 (9.4)	0.0074	0.63
DXA leg fat (kg)	9.1 (3.4)	9.8 (3.4)	9.7 (3.3)	10.0 (3.5)	10.0 (3.6)	0.057	0.63
DXA trunk fat (kg)	12.6 (4.9)	14.0 (5.1)	13.7 (4.6)	14.5 (5.0)	14.9 (5.1)	0.0013	0.83
DXA trunk/leg ratio	1.40 (0.36)	1.46 (0.41)	1.457 (0.40)	1.50 (0.43)	1.54 (0.39)	0.026	0.59
MRI SAT (cm^2)	225.7 (63.9)	235.7 (102.1)	261.4 (109.8)	287.7 (115.0)	342.7 (146.0)	0.0019	0.49
MRI VAT (cm^2)	80. 2 (42.8)	87.4 (53.1)	98. 6 (47.4)	100.4 (49.9)	106.8 (47.6)	0.050	0.90
MRI VAT/SAT ratio	0.37 (0.21)	0.39 (0.22)	0.40 (0.16)	0.36 (0.16)	0.32 (0.11)	0.38	0.59

Table S6. Mean and SD for different indices of obesity given in quintiles of the phthalate metabolite MiBP in men.

	**Q1**	**Q2**	**Q3**	**Q4**	**Q5**		
**Variable**	**Mean (SD)**	**Mean (SD)**	**Mean (SD)**	**Mean (SD)**	**Mean (SD)**	**p-value linear**	**p-value quadratic**
BMI (kg/m^2)	27.3 (3.8)	27.5 (3.9)	26.6 (3. 9)	26.7 (3.6)	26.8 (3.3)	0.088	0.23
Waist (cm)	95.6 (10.3)	96.2 (10.6)	93.0 (11.6)	93.9 (9.6)	94.9 (10.0)	0.19	0.065
Waist/hip ratio	0.96 (0.06)	0.95 (0.07)	0.93 (0.06)	0.94 (0.06)	0.95 (0.06)	0.20	0.0011
DXA total fat (kg)	24.0 (7.6)	24.0 (7.8)	22.5 (9.7)	23.2 (7.7)	23.5 (8.0)	0.34	0.18
DXA leg fat (kg)	6.4 (2.3)	6.5 (2.3)	6.4 (3.5)	6.0 (2.3)	6.4 (2.5)	0.41	0.69
DXA trunk fat (kg)	14.5 (4.8)	14.4 (4.8)	13.4 (5.9)	14.2 (4.9)	14.2 (5.0)	0.38	0.11
DXA trunk/leg ratio	2.35 (0.58)	2.29 (0.54)	2.18 (0.62)	2.43 (0.59)	2.30 (0.62)	0.93	0.21
MRI SAT (cm^2)	225.3 (93.3)	186.7 (77.7)	170.0 (81.2)	189.3 (73.0)	193.3 (28.7)	0.10	0.16
MRI VAT (cm^2)	128.0 (64.2)	133.7 (81.5)	106.8 (60.8)	116.5 (51.3)	113.3 (47.3)	0.30	0.98
MRI VAT/SAT ratio	0.60 (0.27)	0.72 (0.27)	0.64 (0.28)	0.63 (0.21)	0.57 (0.16)	0.82	0.24

Table S7. Mean and SD for different indices of obesity given in quintiles of the phthalate metabolite MMP in women.

	**Q1**	**Q2**	**Q3**	**Q4**	**Q5**		
**Variable**	**Mean (SD)**	**Mean (SD)**	**Mean (SD)**	**Mean (SD)**	**Mean (SD)**	**p-value linear**	**p-value quadratic**
BMI (kg/m^2)	27.1 (4.9)	26. 6 (4.9)	27.0 (4.5)	27.5 (5.5)	27.3 (4.6)	0.10	0.42
Waist (cm)	87.4 (11.8)	86.4 (12.2)	87.2 (10.4)	88.0 (11.9)	89.2 (11.6)	0.041	0.26
Waist/hip ratio	0.86 (0.06)	0.86 (0.07)	0.86 (0.05)	0.87 (0.06)	0.87 (0.06)	0.047	0.65
DXA total fat (kg)	27.7 (9.9)	26.5 (9.5)	28.2 (8.0)	27.1 (9.0)	29.1 (9.7)	0.078	0.25
DXA leg fat (kg)	9.9 (3.7)	9.3 (3.4)	10.0 (3.1)	9.4 (3.2)	10.1 (3.7)	0.39	0.29
DXA trunk fat (kg)	13.8 (5.4)	13.2 (5.1)	14.1 (4.3)	13.7 (4.8)	15.0 (5.3)	0.016	0.21
DXA trunk/leg ratio	1.44 (0.46)	1.45 (0.40)	1.44 (0.33)	1.49 (0.40)	1.53 (0.39)	0.029	0.61
MRI SAT (cm^2)	250.7 (97.3)	225.9 (101.5)	286.8 (106.0)	292.2 (127.2)	234.3 (66.6)	0.065	0.90
MRI VAT (cm^2)	88.9 (51.2)	95.5 (50.1)	96.1 (40.8)	97.6 (57.1)	86.7 (58.6)	0.44	0.41
MRI VAT/SAT ratio	0.37 (0.24)	0.43 (0.19)	0.36 (0.14)	0.34 (0.12)	0.35 (0.15)	0.28	0.31

Table S8. Mean and SD for different indices of obesity given in quintiles of the phthalate metabolite MMP in men.

	**Q1**	**Q2**	**Q3**	**Q4**	**Q5**		
**Variable**	**Mean (SD)**	**Mean (SD)**	**Mean (SD)**	**Mean (SD)**	**Mean (SD)**	**p-value linear**	**p-value quadratic**
BMI (kg/m^2)	26.4 (3.5)	26.6 (3.4)	27.0 (4.0)	27.7 (4.2)	27.1 (3.3)	0.018	0.20
Waist (cm)	92.9 (9.8)	93.7 (10.6)	94.9 (10.8)	96.6 (11.1)	95.3 (9.8)	0.0114	0.22
Waist/hip ratio	0.93 (0.07)	0.93 (0.07)	0.95 (0.06)	0.96 (0.06)	0.95 (0.06)	0.0082	0.25
DXA total fat (kg)	22.9 (8.4)	23.8 (9.1)	22.7 (7.8)	24.1 (7.6)	23.5 (8.2)	0.32	0.53
DXA leg fat (kg)	6.4 (3.1)	6.4 (2.6)	6.1 (2.4)	6.4 (2.4)	6.3 (2.5)	0.92	0.98
DXA trunk fat (kg)	13.7 (5.4)	14.3 (5.6)	13.7 (4.8)	14.7 (4.6)	14.2 (5.1)	0.21	0.44
DXA trunk/leg ratio	2.24 (0.60)	2.24 (0.50)	2.30 (0.52)	2.44 (0.66)	2.33 (0.67)	0.055	0.37
MRI SAT (cm^2)	195.2 (86.7)	174. 4 (61.5)	185.6 (91.4)	201.6 (87.5)	203.2 (76.0)	0.67	0.10
MRI VAT (cm^2)	110.6 (52.5)	122.8 (70.7)	107.6 (44.6)	138.3 (77.8)	106.1 (57.3)	0.37	0.80
MRI VAT/SAT ratio	0.60 (0.25)	0.68 (0.25)	0.62 (0.23)	0.71 (0.28)	0.52 (0.17)	0.58	0.12
